# Elementary Instruction in Chemical Analysis

**Published:** 1844-01

**Authors:** 


ART. IV.—*Elementary Instruction in Chemical Analysis.* By Dr. C. REMIGIUS FRESENIUS, Chemical Assistant in the Laboratory of the University of Giessen. Translated by J. LLOYD BULLOCK, Member of the Chemical Society, &c.—*London,* 1843. 8vo, pp. 284.

IT must be well known to our readers that the Giessen Laboratory is now the resort of students in chemical science, from all countries; on account both of the high reputation of the illustrious philosopher by whom it is superintended, and of the admirable arrangements of the school itself. And as its pupils are for the most part men who are not content with a smattering of knowledge but who enter upon their course of study in good earnest, with the view of qualifying themselves for the successful prosecution of the science in after life; it is obvious that no ordinary opportunities of ascertaining the best plan of instruction, must be enjoyed by those who have the direction of the practical course. As Dr. Fresenius well remarks, "Many ways may lead to the desired end; but one of them will invariably prove the shortest." Along that which his experience has proved to be the shortest cut, although very far from being a *royal road,* Dr. Fresenius endeavours to lead the student, by means of this treatise, which has already gone through two editions in Germany, and which has received the following *imprimatur* from Professor Liebig:

"Dr. Fresenius conducts the course of elementary instruction, in mineral analysis, in the laboratory of the university of Giessen. During the two last sessions he has followed the method described in his work, entitled 'Elementary Instruction in Qualitative Chemical Analysis' This method I can confidently recommend from my own personal experience, to all who are desirous of obtaining instruction in inorganic analysis, for its simplicity, usefulness, and the facility with which it may be apprehended. I consider Dr. Fresenius' work extremely useful as an introduction to Professor H. Rose's excellent Manual, and for adoption in institutions where practical chemistry is taught, but it is especially adapted to the use of pharmaceutical chemists. Further, a number of experiments and discoveries have been recently made in our laboratory, which have enabled Dr. Fresenius to give many new and simplified methods of separating substances, which will render his work equally welcome to those who are already familiar with the larger works on inorganic analysis." (Preface.)

A treatise of this kind is obviously not adapted for notice in detail; but we shall give an extract from the editor's preface, which will indicate the leading features of the work, and especially the points on which it chiefly differs, as regards its plan, from that of Mr. Parnell, to which we directed the attention of our readers not long since.

"The author was led to compose this volume upon perceiving that the larger works on chemical analysis, such as H. Rose's, Duflos', and others, although admirable in themselves, present great difficulties to beginners, which difficulties may be summed up under three heads; 1st, Too great copiouness and detail; 2d, the absence of explanations of the causes of phenomena, i. e. the *theory* of the operations and reactions; and, 3d, the omission altogether of many substances of very frequent occurrence, especially in the operations of the pharmaceutist, such as the organic acids, &c. In avoiding these objections to former works on chemical analysis, Dr. Fresenius, I think, is not chargeable with having fallen into the opposite extreme of being too concise or elementary.

"The student may perhaps at first be disappointed in taking up this work to find that there are no tables constructed to furnish him at a glance with all he is desirous to know of tests and reactions, and to save him, as he may think, trouble and time. But this has not arisen from oversight; the question of the advantage or disadvantage of tables to the students has been fully considered; and the author has decided,—and the decision is borne out by the highest authorities,—that such tables serve no really good purpose; they rather, on the contrary, supply but very superficial information, and satisfy the student before they have really informed him. The information contained in this work, like every other professing to teach a practical science, requires application and perseverance to attain; but if begun at the beginning, if the student will carefully go over the necessary preliminary facts, the examination of his tests, and the reaction of the simple bodies consecutively, and make himself master of this very simple and elementary part of the course, he will find no difficulties when entering upon the more elaborate, and—what might appear without this preparation—complex and intricate processes of the second part, the analysis of compound bodies. It is altogether another question whether the student should or should not exercise himself and his memory, by tabulating the results of his experiments as he proceeds; and to this question we reply in the affirmative; but it must be left to individuals to act in this according to their own judgments, and their own feeling of its necessity." (Preface, pp. 6, 7.)

Mr. Bullock appears to us to have executed his task as translator and editor, in a very creditable manner; so that the work altogether wears quite an English garb. We would, however, ask him why he has omitted the word "qualitative" from the original designation of the treatise? The title, as it now stands, is very liable to mislead others, as it has ourselves; since the book will undoubtedly be ordered by many, who expect that a treatise emanating from the school of Giessen, shall include *organic* as well as inorganic analysis. With the exception of the organic acids, the substances examined in this volume belong altogether to the mineral kingdom. *

